# Rabies viruses leader RNA interacts with host Hsc70 and inhibits virus replication

**DOI:** 10.18632/oncotarget.16517

**Published:** 2017-03-23

**Authors:** Ran Zhang, Chuangang Liu, Yunzi Cao, Muhammad Jamal, Xi Chen, Jinfang Zheng, Liang Li, Jing You, Qi Zhu, Shiyong Liu, Jinxia Dai, Min Cui, Zhen F. Fu, Gang Cao

**Affiliations:** ^1^ State Key Laboratory of Agricultural Microbiology, Huazhong Agricultural University, Wuhan 430070, China; ^2^ College of Veterinary Medicine, Huazhong Agricultural University, Wuhan 430070, China; ^3^ Key Laboratory of Development of Veterinary Diagnostic Products, Ministry of Agriculture, College of Veterinary Medicine, Huazhong Agricultural University, Wuhan 430070, China; ^4^ Department of Pathology, College of Veterinary Medicine, University of Georgia, Athens, GA 30602, USA; ^5^ Department of Physics and Key Laboratory of Molecular Biophysics of the Ministry of Education, Huazhong University of Science and Technology, Wuhan 430074, China

**Keywords:** leader RNA (LeRNA), rabies virus (RABV), Hsc70, post-exposure prophylaxis (PEP), RNA-Protein interaction

## Abstract

Viruses have been shown to be equipped with regulatory RNAs to evade host defense system. It has long been known that rabies virus (RABV) transcribes a small regulatory RNA, leader RNA (leRNA), which mediates the transition from viral RNA transcription to replication. However, the detailed molecular mechanism remains enigmatic. In the present study, we determined the genetic architecture of RABV leRNA and demonstrated its inhibitory effect on replication of wild-type rabies, DRV-AH08. The RNA immunoprecipitation results suggest that leRNA inhibits RABV replication via interfering the binding of RABV nucleoprotein with genomic RNA. Furthermore, we identified heat shock cognate 70 kDa protein (Hsc70) as a leRNA host cellular interacting protein, of which the expression level was dynamically regulated by RABV infection. Notably, our data suggest that Hsc70 was involved in suppressing RABV replication by leader RNA. Finally, our experiments imply that leRNA might be potentially useful as a novel drug in rabies post-exposure prophylaxis. Together, this study suggested leRNA in concert with its host interacting protein Hsc70, dynamically down-regulate RABV replication.

## INTRODUCTION

During the last two decades, tremendous evidence has unveiled regulatory non-coding RNA (ncRNA) as a leading actor in the RNA world throughout nearly all organisms ranging from viruses to humans [1]. In eukaryotes, regulatory ncRNAs can be divided into two classes: one is small ncRNA consisting of short interfering RNA (siRNA), microRNA (miRNA) and PIWI-interacting RNA (piRNA), the other is long ncRNAs (lncRNA) which is poorly conserved [2]. These ncRNAs participate in most of the major cellular processes including chromatin modification, transcriptional regulation and post-transcriptional regulation through diverse mechanisms, such as regulating chromatin structure, modulating RNA maturation and transport as well as controlling protein synthesis [2–6]. Although the role of eukaryotic ncRNA has been well documented, little is known about the molecular function of viral ncRNA. Viral ncRNA has been mostly studied in DNA viruses, especially herpesviruses [2, 7]. Several positive-strand RNA viruses, such as human immunodeficiency virus (HIV), Dengue virus (DENV) and West Nile virus (WNV) can also encode microRNA-like ncRNAs to regulate virus replication [8–11]. Although negative-strand RNA viruses don't produce viral miRNAs, it generates a small ncRNAs termed viral leader RNAs (leRNAs). In this scenario, influenza virus, vesicular stomatitis virus (VSV) and rabies virus (RABV) produce high levels of leRNAs with different length, which play important regulatory roles in virus life cycle [12].

LeRNA has been discovered in RABV for more than 30 years, but its function and the underlying mechanism are still poorly understood [13–16]. RABV is made up of single-stranded, negative-sense, nonsegmented RNA genome and five structure proteins [17, 18]. LeRNA is the first viral product produced in RABV infected cells during infection. Moreover, leRNA is also observed in other members of the *Rhabdoviridae* family including VSV [19]. Various lengths of RABV leRNA have been reported, mostly found to be 56 nt or 58 nt [13]. LeRNAs of RABV are capable of activating dendritic cells (DCs) and its level determines the status of DCs activation [20]. Previous research has proposed that VSV leRNA and VSV nucleoprotein (N) complex play an important role in the transition from viral RNA transcription to replication [21]. Similarly, leRNA and RABV N could also regulate viral RNA transcription and replication [22]. When small RNA performs its function, it often recruits an interacting protein and forms a RNA-protein complex. siRNAs and miRNAs often need to form RNA-induced silencing complexes (RISCs) to fulfil their functions [23]. A member of Argonaute (Ago) family proteins located in the heart of RISC has an important function on target cleavage, and can cleave pre-miRNA to mature miRNA [24, 25]. It has been reported that RABV N protein preferentially interacts with leRNA over other RNA species [26]. La protein is another well-known protein interacting with RABV leRNA [13]. It is also associated with VSV leRNA and norovirus viral RNA [27, 28]. Moreover, in eukaryotic cells, La protein associates with the 3′ terminus of many newly synthesized small RNAs to protect them from exonucleases [29]. Thus, the important role of the interaction between La protein and leRNA deserves further investigation [13]. Although RABV leRNA is a well-known ncRNA transcript of RABV genome and plays important roles in viral transcription and replication, the underlying mechanism remains to be elucidated.

The aim of this study is to investigate the *in vitro* and *in vivo* function of leRNA on RABV replication of DRV-AH08, a wild type RABV strain, and the detailed molecular mechanisms. To this end, we applied aptamer mediated RNA-protein precipitation assay coupled with mass spectrometric analysis and identified a host cell interacting protein of leRNA, Hsc70. This protein has been shown to bind to AU-rich elements (AREs) in the 3′-untranslated region of specific mRNAs to enhance their stability [30] and displays important functions on cellular protein degradation [31], autophagy [32], synaptic signal transduction [33], and notably, loading small RNA duplexes into Argonaute proteins [34]. Through biochemical assay, real time PCR, immunohistochemical staining and *in vivo* experiments, we showed that leRNA regulates RABV replication via interacting with Hsc70 and knockdown of the Hsc70 resulted in the suppression of RABV replication. In addition, our *in vitro* and *in vivo* experiments implied that leRNA might be potentially useful as a novel drug in rabies post-exposure prophylaxis.

## RESULTS

### Architecture of leRNA from RABV DRV-AH08 strain

**Figure 1:**
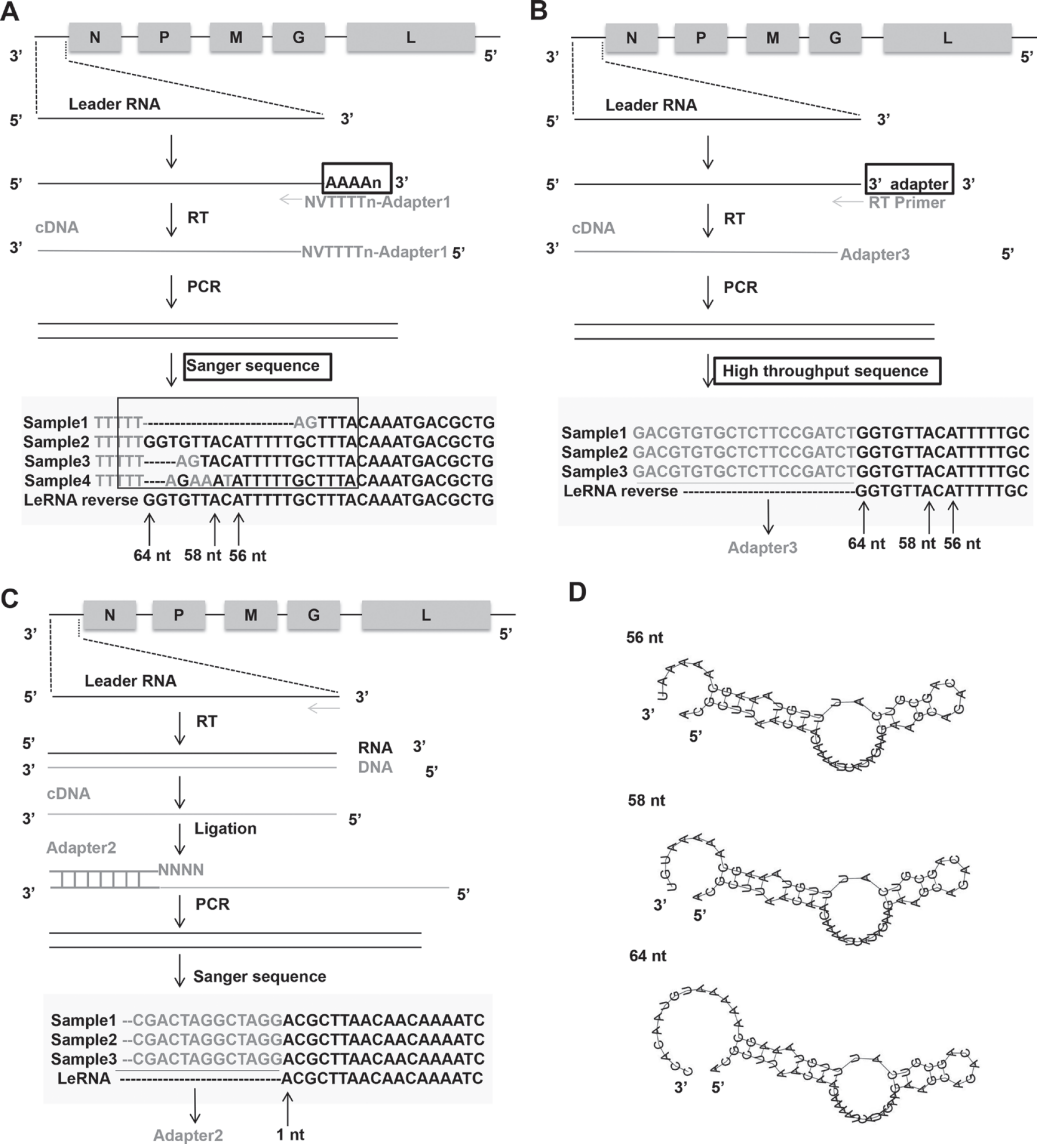
Architecture of leRNA from RABV DRV-AH08 strain (**A**) Construct 3′ end library of leRNA to confirm the transcription end sites of leRNA by Sanger sequencing. Nucleotides in grey mean the unmatched nucleotides between viral genome and leRNA. Sample means a specific clone that was sequenced. The “V” and “N” mean degenerate bases, wherein “V” represents A/G/C, and “N” represents A/T/G/C. (**B**) Another way to construct 3′ end library of leRNA to confirm the transcription end sites of leRNA by high-throughput sequencing. (**C**) Construct 5′ end library of leRNA to confirm the transcription start sites of leRNA. (**D**) Predicted secondary structures of leRNA from RABV DRV-AH08 strain using RNAflod webserver.

To determine the precise sequence and length of leRNA, the total RNAs were extracted from mouse brain infected with RABV DRV-AH08 strain, a wild-type RABV. The RNA was subjected to sequencing of the 3′ and 5′ ends of leRNA. Firstly, the total RNAs were added with Poly(A) tail through Poly(A) Polymerase and reversely transcribed by RT primers containing adapter 1 before oligo d(T) (Figure 1A). Then the cDNA was amplified by primer adapter 1 and primer complementary to the 3′ terminus of the virus genome, to obtain dsDNA, which was inserted into pMD-18T vector to construct the 3′ end library. Sequencing of 30 clones indicate that the transcription of leRNA is ended at multiple sites, resulting in different length from 40 nt to 79 nt, among which 64 nt seems to be the most abundant (40%) (Figure 1A). The length of remaining leRNA varies from 56 nt (17%), 58 nt (10%) and many other sizes.

To further validate the length of leRNA, total RNA was extracted from DRV-AH08-infected SK-N-SH cells at 12 h post-infection, and ligated an adaptor named 3′ SR to the 3′ end of the total RNA. Specific reverse transcript primer complementary with 3′ SR was used for reverse transcription. After PCR amplification by Illumina sequence primer, the DNA fragments were subjected to high throughput sequencing (Figure 1B). Analysis of the results from the high-throughput sequencing revealed that 64 nt long RNA is one of the most prominent variants.

Similarly, for the 5′ end library, cDNA of leRNA was ligated with adapter 2 containing NNNN overhang to match the 3′ terminal of the cDNA (Figure 1C), then amplified and cloned into pMD-18T vector for sequencing. A total of 30 clones were sequenced and found to have the same transcriptional start site of the leRNA from the first base of the 3′ end of virus genome (Figure 1C). It means that different lengths of leRNA are all transcribed from the first base of the 3′ end of virus genome.

Despite the various sizes of these leRNA species, the putative secondary structures predicted by RNAfold webserver (http://rna.tbi.univie.ac.at/cgi-bin/RNAfold.cgi) show a typical hairpin structure for the 56 nt, 58 nt and 64 nt leRNA from DRV-AH08 (Figure 1D).

### Overexpression of leRNA inhibits RABV infection *in vitro* and *in vivo*

**Figure 2:**
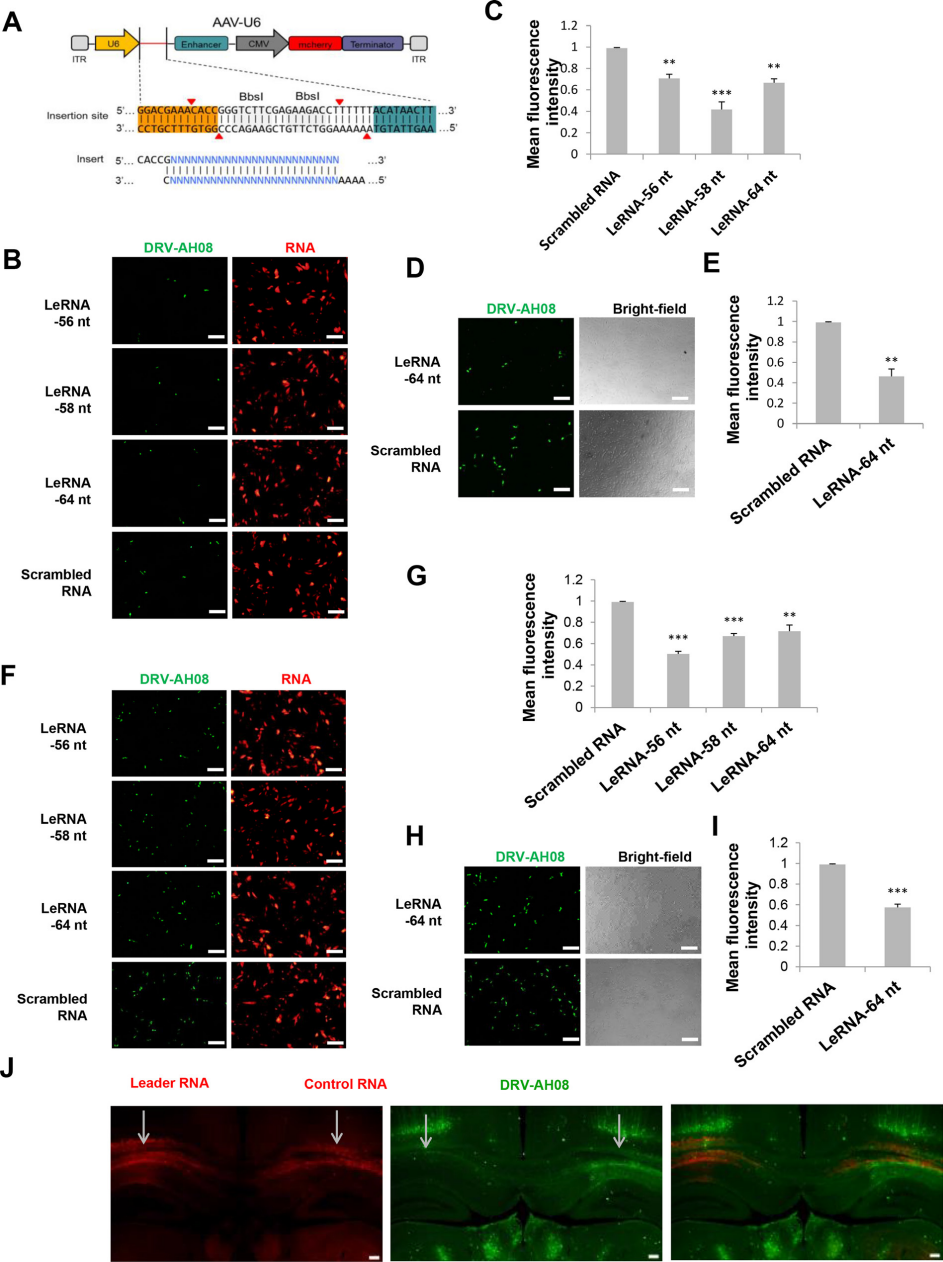
Overexpression of leRNA inhibits RABV infection *in vitro* and *in vivo* (**A**) A schematic diagram of non-coding RNA overexpression vector, pAAV-U6, with two *Bbs*I sites for insertion of non-coding RNA. Two paired oligos of insertion were shown. (**B**) SK-N-SH cells were transfected with pAAV-U6-leRNA-56 nt, pAAV-U6-leRNA-58 nt, pAAV-U6-leRNA-64 nt and pAAV-U6-scrambled RNA respectively and then infected with DRV-AH08 (MOI is 0.01) at 24h after transfection. RABV antigens were detected by immunofluorescence using FITC-conjunct anti-N antibody at 24 h post-infection, mCherry indicates the RNA positive cells. (**C**) Meanfluorescence intensity of DRV-AH08 in Figure 2B was measured by Image-Pro Plus 6.0. (**D**) SK-N-SH cells were transfected with *in vitro* synthesized leRNA and scrambled RNA, respectively, and then infected with DRV-AH08 (MOI is 0.01). The DRV-AH08 was detected by immunofluorescence staining using FITC-conjunct anti-N antibody at 24 h post-infection. (**E**) Meanfluorescence intensity of DRV-AH08 infection in Figure 2D was analyzed by Image-Pro Plus 6.0. (**F**) SK-N-SH cells were transfected with pAAV-U6-leRNA-56 nt, pAAV-U6-leRNA-58 nt, pAAV-U6-leRNA-64 nt and pAAV-U6-scrambled RNA respectively and then infected with DRV-AH08 (MOI is 0.1) at 24 h after transfection. RABV antigens were detected by immunofluorescence using FITC-conjunct anti-N antibody at 24 h post-infection. mCherry indicates the RNA positive cells. (**G**) Meanfluorescence intensity of DRV-AH08 in Figure 2F was measured by Image-Pro Plus 6.0. (**H**) SK-N-SH cells were transfected with *in vitro* synthesized leRNA and scrambled RNA, respectively, and then infected with DRV-AH08 (MOI is 0.1). The DRV-AH08 was detected by immunofluorescence staining using FITC-conjunct anti-N antibody at 24 h post-infection. (**I**) Meanfluorescence intensity of DRV-AH08 infection in Figure 2H was analyzed by Image-Pro Plus 6.0. (**J**) Mice were injected with rAAV-U6-leRNA-64 nt (red) or rAAV-U6-control RNA (red) into left and right hippocampus respectively, and then challenged with DRV-AH08 after two weeks. DRV-AH08 infection was detected by immunofluorescence staining using mouse monoclonal anti-P antibody and Alexa Fluor 488 IgG (green). All of the scale bars are 200 μm. All experiments were repeated at least three times. Data are presented by mean ± SEM. *P* values were determined by Student's *t-test*. *P < 0.05; **P < 0.01; ***P < 0.001.

To investigate leRNA function during RABV infection, leRNA of DRV-AH08 (56 nt, 58 nt and 64 nt) and scrambled RNA (64 nt) were cloned into pAAV-U6 between *BbsI* sites and overexpressed under U6 promoter with red fluorescence protein mCherry as an indicator. The sequential TTTTT is the terminator of U6 promoter (Figure 2A). As shown in Figure 2B and 2C, all of the leRNA (red) significantly reduced the number of virus positive cells (green) as compared with that of the scrambled RNA 24 h post-infection with DRV-AH08 at a MOI of 0.01. To further confirm the inhibitory function of leRNA, *in vitro* synthesized 64 nt leRNA was directly transfected into SK-N-SH cells and then infected with DRV-AH08 at a MOI of 0.01. Similarly, significantly fewer virus positive cells were observed in cells transfected with leRNA than in those with scrambled RNA (Figure 2D and 2E). After increasing the virus dose to a MOI of 0.1, the amount of RABV positive cells was still significantly reduced in SK-N-SH cells transfected with small RNA-expressing plasmids (Figure 2F and 2G), or with *in vitro* transcribed leRNA (Figure 2H and 2I) than with scrambled RNA.

To determine if the leRNA has the same inhibitory function *in vivo*, more than six mice were simultaneously injected with rAAV-U6-leRNA AAV virus and rAAV-U6-control RNA AAV virus (control RNA was an irrelevant RNA with hairpin structure, of which the sequence was described in *material and methods*) in the left and right hippocampus, respectively, followed by challenge with DRV-AH08. The injection of AAV viruses expressing leRNA or control RNA were indicated by the mCherry fluorescence in the hippocampus (Figure 2J). In the left hippocampus injected with leRNA-AAV virus, RABV (green) was remarkably less than that in the right hippocampus injected with the control RNA-AAV virus. Taken together, leRNA could negatively regulate RABV DRV-AH08 infection both *in vitro* and *in vivo*.

### LeRNA inhibits RABV replication by interfering the binding of RABV nucleoprotein with genomic RNA

**Figure 3:**
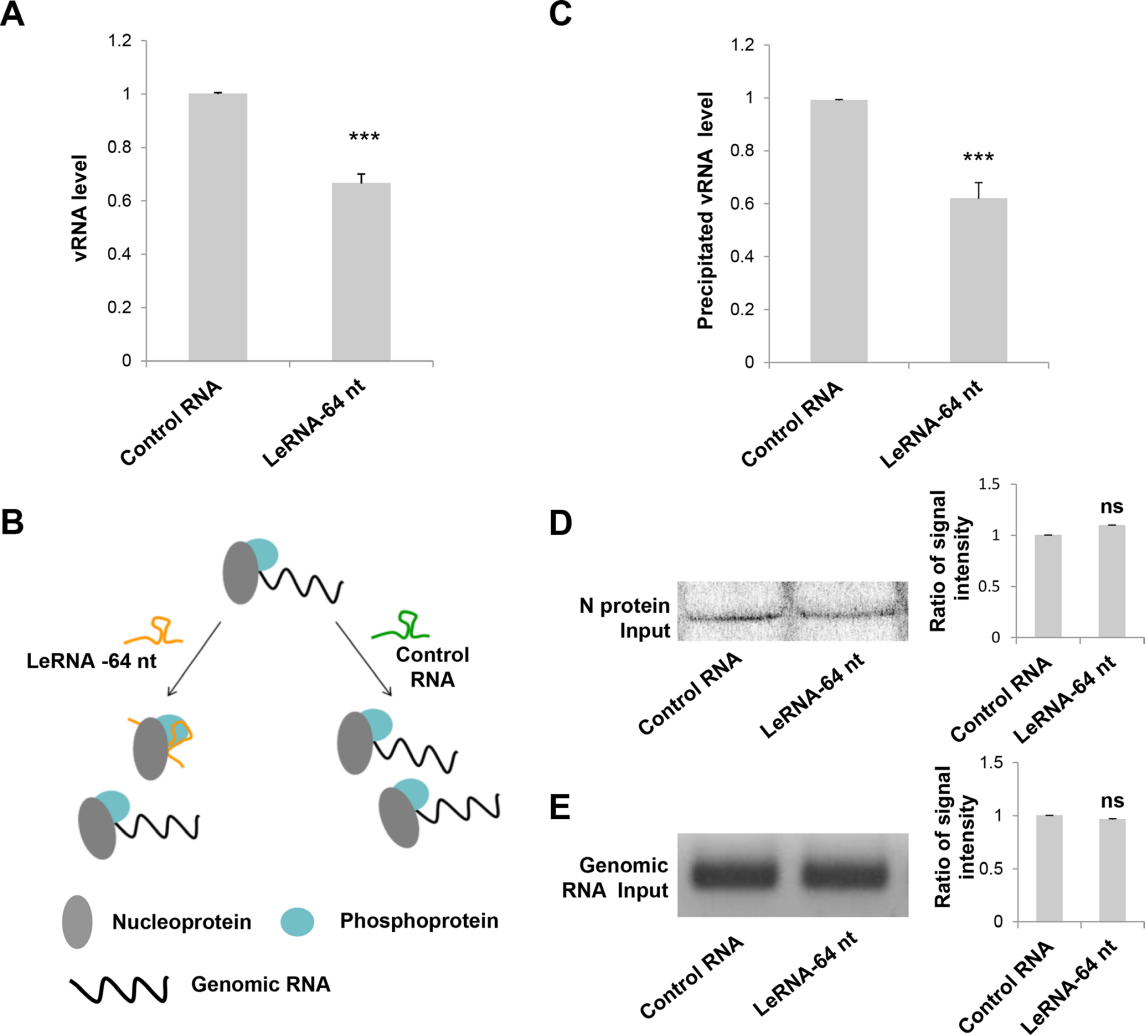
LeRNA inhibits RABV replication by interfering the binding of RABV nucleoprotein to genomic RNA (**A**) SK-N-SH cells were infected with rAAV-U6-leRNA-64 nt or rAAV-U6-control RNA AAV virus and after 24 h, infected with DRV-AH08 (MOI is 0.01). The levels of DRV-AH08 viral genomic RNA (vRNA) were detected by Real Time PCR at 12 h post-infection with DRV-AH08. (**B**) Schematic diagram of the precipitation (IP) of nucleoprotein to genomic RNA under leRNA and control RNA overexpression respectively. (**C**) N protein with Fc, P protein and DRV-AH08 genome fragment were co-transfected with leRNA or control RNA in HEK-293T cells, the level of N protein captured genomic RNA was determined by Real Time PCR after 48 h transfection. The ratio of precipitated RNA/Input RNA of leRNA group has been normalized to the ratio of control RNA group. (**D**) The input of N protein in precipitation assay were detected by Western blot. The ratio of signal intensity of input N protein was quantified using ImageJ. (**E**) The input of viral genomic RNA in precipitation assay were detected by RT-PCR. The ratio of signal intensity of input genomic RNA were quantified using ImageJ. All experiments were repeated at least six times. All values are the mean ± SEM. *P* values were determined by Student's *t-test*. *P < 0.05; **P < 0.01; ***P < 0.001.

The decrease of RABV infection by leRNA could be due to the inhibition of RABV replication in the host cells. To test this hypothesis, the level of virus genomic RNA (vRNA) was measured in SK-N-SH cells infected with DRV-AH08 12 h and 30 h post-infection in the presence of rAAV-U6-leRNA AAV virus or rAAV-U6-control RNA AAV virus. The level of vRNA was significantly decreased by leRNA overexpression as compared with that by control RNA (40%) at 12 h (Figure 3A) and 30 h (Supplementary Figure 1) post-infection, indicating that leRNA inhibits RABV replication.

As it has been shown that RABV N protein binds preferentially to the leRNA than the genomic RNA [26], we investigated whether leRNA inhibits virus replication by interfering with the interaction between the N protein and the genomic RNA. To this end, N protein with Fc tag, P protein and DRV-AH08 genomic fragment (1-1514 bp) were co-expressed with the leRNA or control RNA in HEK-293T cells. The protein-RNA pull down assay showed that the precipitated genomic RNA by N protein is significantly reduced (by 40%) in leRNA overexpression group, compared with that in the control RNA group (Figure 3C). Meanwhile, the total N protein (Figure 3D) and genomic RNA (Figure 3E) were equivalent between the leRNA group and the control RNA group. These data suggest that leRNA may compete with genomic RNA to bind with N protein and thus inhibits RABV replication (Figure 3B).

### LeRNA interacts with host protein Hsc70

**Figure 4:**
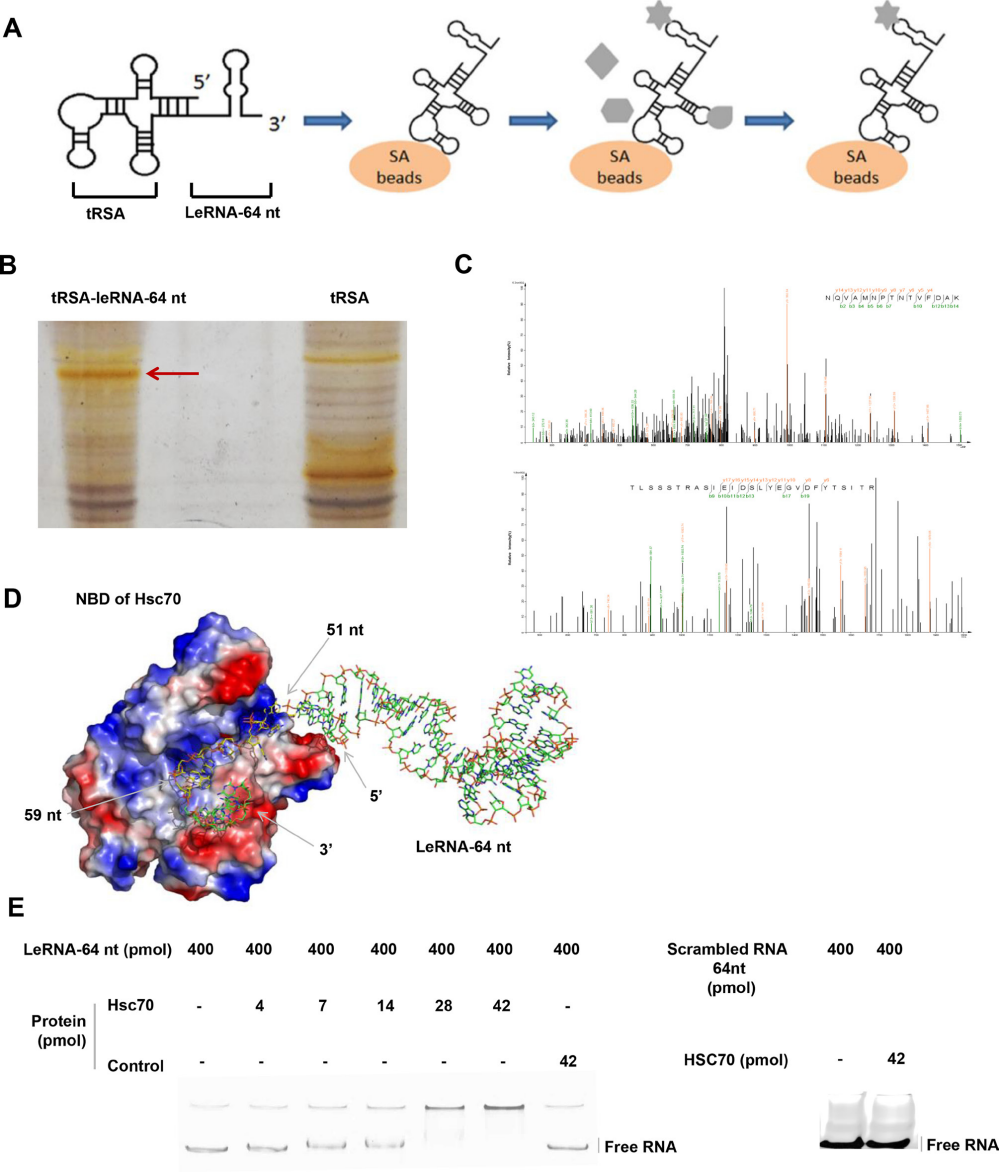
LeRNA interacts with host protein Hsc70 (**A**) Schematic diagram of tRSA aptamer mediated RNA-protein precipitation assay. (**B**) Identification of host protein interacting with the leRNA-64 nt of DRV-AH08. The tRSA alone coupled to streptavidin beads was referred as control, and the captured proteins were eluted and analyzed by SDS–PAGE with silver staining. The position of proteins for mass spectrometry analysis in the leRNA-64 nt compared to the control are highlighted with arrowheads. (**C**) Identification of Hsc70 by LC-MS/MS. (**D**) A putative 3D structure of Hsc70 nucleotide binding domain and leRNA complex. (**E**) Confirmation of the interaction between Hsc70 and leRNA by EMSA. The leRNA-64 nt (400 pmol) were incubated with increased concentrations of Hsc70 (0, 4, 7, 14, 28, and 42 pmol) and analyzed on 8% w/v polyacrylamide non-denaturing gel. The irrelevant protein 4BOW was referred as control protein. Right panel, 42 pmol control protein incubated with leRNA-64 nt (400 pmol). The scrambled RNA (400 pmol) were incubated with Hsc70 (42 pmol) and analyzed on 8% w/v polyacrylamide non-denaturing gel. RNAs were stained using SYBR^®^ Gold Nucleic Acid Gel Stain.

As leRNA plays a crucial role in down-regulating RABV replication, we next investigated if any host factor binds to RABV leRNA. To this end, tRSA aptamer (tRNA scaffold to a Streptavidin aptamer) [35] mediated RNA-protein precipitation assay was performed to screen proteins from the host that could bind to leRNA. Firstly, the *in vitro* transcribed tRSA-leRNA was mixed with SA beads (Streptavidin beads), followed by incubation with mouse brain lysates (Figure 4A). After SDS–PAGE electrophoresis and silver staining, the captured proteins presented in tRSA-leRNA group (red arrow) but not in tRSA control group were subjected to mass spectrometry (Figure 4B). By this approach, Hsc70 was identified as one of the leRNA-interacting proteins in the host (Figure 4C) (Two independent Hsc70 Peptides were identified. NQVAMNPTNTVFDAK and TLSSSTRASIEIDSLYEGV DFYTSITR). In order to confirm the interaction of Hsc70 with RABV leRNA, we analysed the mobility shifting of purified leRNAs in the presence of Hsc70 or irrelevant control protein 4BOW (an irrelevant control protein from Zobellia galactanivorans) by EMSA assay. With gradually increasing the amount of Hsc70 from 4 pmol, the shift of leRNA is clearly restrained to loading slot whereas the mobility of leRNA was not affected by 4BOW even in high concentrations (Figure 4E). We also verified the interaction of scrambled RNA with Hsc70 by EMSA and found that Hsc70 scarcely binds to scrambled RNA (Figure 4E). To further understand the details of the Hsc70 and leRNA interaction, the 3D structure of Hsc70 nucleotide docking was modelled with leRNA. The 3D structure of Hsc70 (PDB 3hsc) was obtained from Protein Data Bank (PDB) [36, 37] and the predicted 3D structure of leRNA by RNA Composer web server [38]. The putative RNA structure and the protein Hsc70 structure were then docked to generate RNA-protein complex decoys by 3dRPC [39] with default parameters. As shown in Figure 4D, leRNA interacts with Hsc70 nucleotide-binding pocket located at the N-terminal nucleotide binding domain (NBD) through its sequence from site 51 nt to site 59 nt.

### Knockdown of host Hsc70 up-regulates leRNA level and inhibits RABV replication

**Figure 5:**
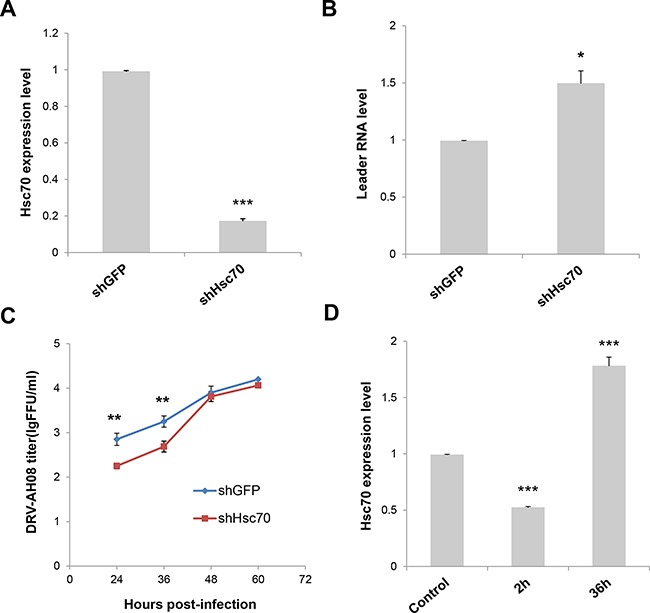
Knockdown of Host Hsc70 up-regulates leRNA level and inhibits RABV replication (**A**) Knockdown of Hsc70 by rAAV-U6-shHsc70 in SK-N-SH cells. (**B**) SK-N-SH cells were infected with rAAV-U6-shHsc70 and rAAV-U6-shGFP AAV virus respectively and then infected with DRV-AH08 (MOI is 0.01). LeRNA expression were detected by Real Time PCR at 12 h post-infection with DRV-AH08. (**C**) SK-N-SH cells were infected with rAAV-U6-shHsc70 and rAAV-U6-shGFP AAV virus respectively and infected with DRV-AH08 (MOI is 0.01) 12 h later. Viral titers were measured by FITC-conjunct anti-N antibody from 24 hours to 60 hours post-infection of DRV-AH08. (**D**) Analysis of Hsc70 expression in SK-N-SH cells at 2 h and 36 h post-infection of DRV-AH08 (MOI is 0.01) by Real Time PCR, the level of Hsc70 has been normalized to uninfected group respectively. All experiments were repeated at least six times. Data are presented by mean ± SEM. *P* values were determined using Student's *t-test*. *P < 0.05; **P < 0.01; ***P < 0.001.

To investigate the function of Hsc70 on leRNA and RABV replication, the expression of Hsc70 RNA was knocked down through RNA interference and the leRNA level was analyzed in SK-N-SH cells infected by DRV-AH08 12 h post-infection. As shown in Figure 5A, the expression level of Hsc70 was largely decreased by shHsc70 but not by shGFP, resulting in a significant increase in the level of leRNA (Figure 5B). Moreover, viral titer was significantly reduced at 24 and 36 h post-infection when Hsc70 RNA was knocked down (Figure 5C). These data show the influence of Hsc70 on the level of leRNA and RABV replication. To test whether RABV infection could regulate Hsc70 in turn, Real Time PCR was performed to detect Hsc70 RNA level after DRV-AH08 infection. Infection of DRV-AH08 resulted in about 50% reduction of host Hsc70 RNA level at 2 h post-infection, but a significant increase at 36 h post-infection versus uninfected control (Figure 5D), suggesting a dynamic regulation of Hsc70 at different stages during RABV infection. Notably, we demonstrated that the GAPDH expression level was not effected by RABV infection (Supplementary Figure 2).

### Hsc70 plays important role in the positive regulation of RABV replication by leRNA

**Figure 6:**
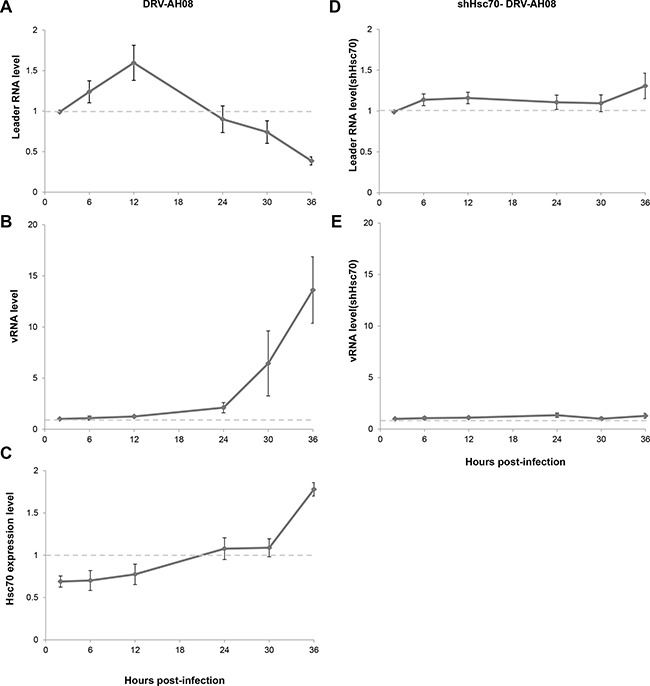
Hsc70 plays important role in the regulation of RABV replication by leRNA (**A**–**C**) LeRNA level (A), viral genomic RNA level (B) and Hsc70 mRNA level (C) in SK-N-SH cells from 2 h to 36 h after DRV-AH08 infection (MOI is 0.01). (**D**, **E**) SK-N-SH cells were transfected Hsc70 shRNA and then infected with DRV-AH08 (MOI is 0.01). LeRNA level (D) and viral genomic RNA level (E) were detected by Real Time RT-PCR from 2 h to 36 h after DRV-AH08 infection. All experiments were repeated at least nine times.

As the expression of host Hsc70 changes dynamically during RABV infection, it could potentially influence the level of leRNA and consequently RABV replication. We further investigated the expression of leRNA, Hsc70 and RABV vRNA at different time points after DRV-AH08 infection. Real Time PCR experiments revealed that leRNA was detected 2 h post-infection, and increased rapidly to a maximal level at 12 h then reduced gradually (Figure 6A), whereas the virus genomic RNA maintained at a low level in the early stage but increased from 12 h and rised sharply from 24 h post-infection (Figure 6B). During DRV-AH08 infection, the Hsc70 level was reduced at the early stage, followed by a steady increase from 12 h post-infection (Figure 6C). Thus, there is tight correlation among the expression of host Hsc70, leRNA and viral genomic RNA. When Hsc70 was knocked down by shHsc70 in SK-N-SH cells, the level of leRNA maintained at a constant level from 2–36 h post-infection. Correspondently, the level of viral genomic RNA was stable during the first 36 h post-infection (Figure 6D and 6E). Taken together, RABV infection dynamic changes the level of host Hsc70, which correlates negatively with leRNA but positively with viral genomic RNA. Knockdown of Hsc70 demolished the dynamic changes in the level of leRNA and virus genomic RNA during the first 36 h post infection, suggesting that Hsc70 may play an important role in the positive regulation of RABV replication by leRNA.

### LeRNA could be potentially used in rabies post-exposure prophylaxis

**Figure 7:**
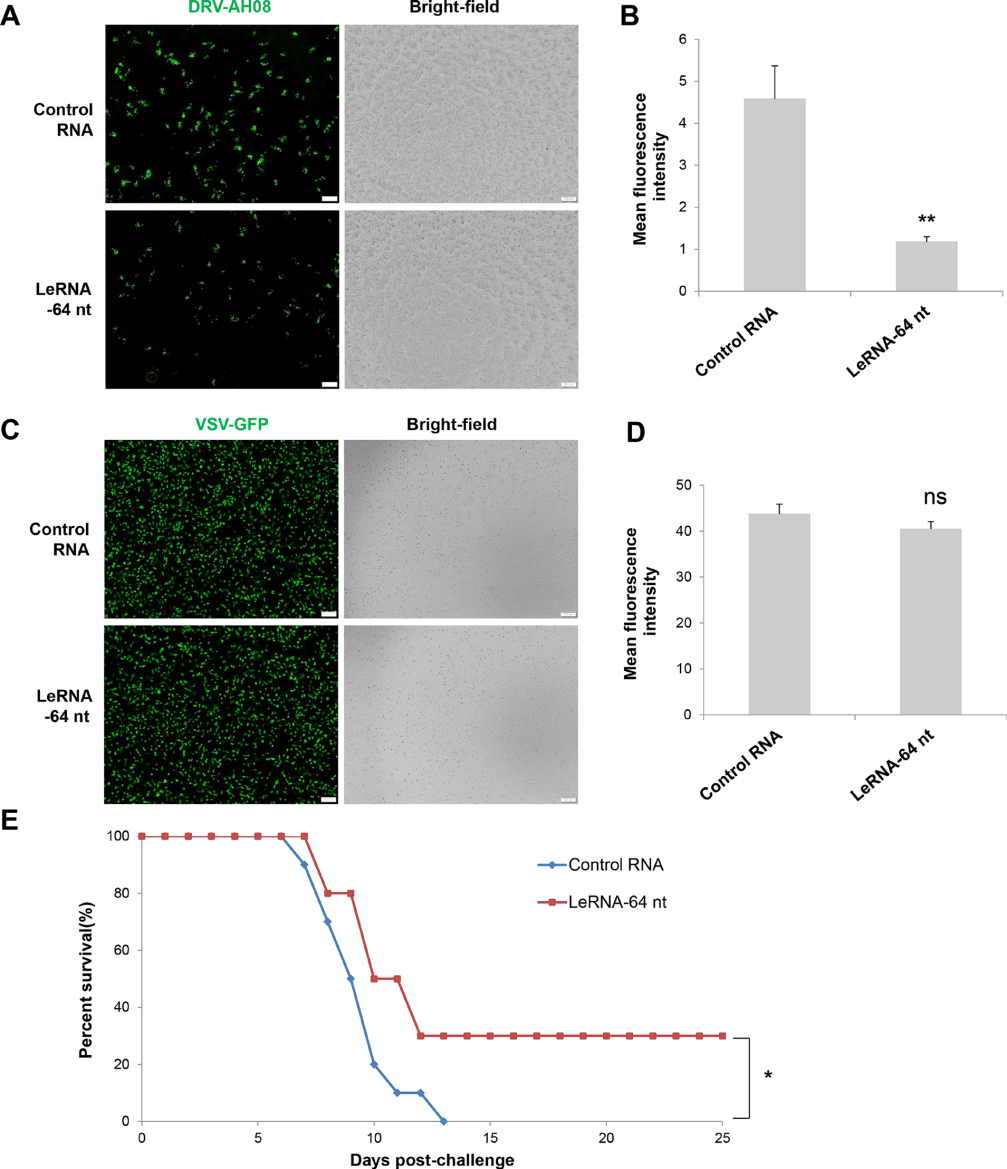
LeRNA could be potentially used in rabies post-exposure prophylaxis (**A**) SK-N-SH cells were infected with DRV-AH08 (MOI is 0.01), and then transfected with *in vitro* synthesized leRNA-64 nt mimics and control RNA 1 h later. Viral replication was detected by immunofluorescence using FITC-conjunct anti-N antibody at 24 h post-infection. All of the scale bars are 200 μm. (**B**) Mean fluorescence intensity measurements of DRV-AH08 infection in Figure 7A by Image-Pro Plus 6.0. All experiments were repeated at least three times. Data are presented by mean ± SEM. *P* values were determined by Student's *t-test*. *P < 0.05; **P < 0.01. (**C**) SK-N-SH cells were infected with VSV-GFP (MOI is 0.01), and then transfected with *in vitro* synthesized leRNA-64 nt and control RNA 1 hour later. Viral replication was detected by immunofluorescence microscopy detecting the GFP at 24 h post-infection. All of the scale bars are 200 μm. (**D**) Mean fluorescence intensity measurements of VSV-GFP infection in Figure 7C by Image-Pro Plus 6.0. All experiments were repeated at least three times. Data are presented by mean ± SEM. *P* values were determined by Student's *t-test*. *P < 0.05; **P < 0.01. (**E**) Survival rates of mice with intramuscular injection with 10^4^ FFU of DRV-AH08 (*n* = 10) followed by rAAV-U6-leRNA-64 ntAAV virus and rAAV-U6-control RNA AAV virus 1 h later. The survival rates were analyzed for statistical significance by Kaplan-Meier plots (*n* = 10 in each group; *P <* 0.05 by log-rank test).

Considering the inhibitory effect of leRNA on RABV replication, it is of great necessity to explore the potential application of leRNA in rabies post-exposure prophylaxis. To this end, SK-N-SH cells were firstly infected by DRV-AH08, and then transfected with *in vitro* synthesized leRNA or control RNA 1 h post infection. VSV, another member of the *Rhabdoviridae* family, was used as a control. At 24 h post infection, significantly fewer virus positive cells were observed in leRNA-treated cells as compared with the control RNA-treated cells (Figure 7A). The mean fluorescent intensity (MFI) of RABV in leRNA- treated cells was significantly decreased compared with that of the control RNA-treated cells (Figure 7B). Importantly, VSV genome replication did not display significant difference from cells treated with RABV leRNA or control RNA (Figure 7C and 7D). Thus, leRNA can specifically and effectively inhibit RABV replication post-infection. To determine if leRNA can be used to inhibit RABV replication post-exposure *in vivo*, mice were challenged intramuscularly with a lethal dose of DRV-AH08 (10^4^FFU per mouse), followed by rAAV-U6-leRNA AAV virus or rAAV-U6-control RNA AAV virus injection in the same site 1 h post-exposure respectively. The clinical signs and death were monitored for 25 days. As shown in Figure 7E, all mice injected with control RNA AAV virus were dead by 13 day post infection. In contrast, 30% of mice injected with leRNA AAV virus survived for more than 25 days, indicating the protective effect of leRNA *in vivo*. Taken together, leRNA could inhibit DRV-AH08 replication post-infection both *in vitro* and *in vivo*, implying a potential application of leRNA in rabies post-exposure prophylaxis.

## DISCUSSION

During the endless arms’ race between the host and the virus, both belligerent parties have equipped ncRNA to fight with each other. While the role of host ncRNA during antiviral defense has been well documented, little is known about the molecular function of viral ncRNA during infection. It has long been known that negative-strand RNA viruses, such as VSV and RABV, transcribe a small RNA from the 3′ end of their genome [13, 40]. Previous studies have shown that leRNAs transcribed by several VSV serotypes range from 50 to 53 nt in length [40]. For RABV, Kurilla MG et al. reported that leRNAs in RABV infected BHK-21 cells have different lengths, usually terminating at positions 55 through 58 nt [13]. In addition, the termination positions at 43 nt and 60 nt have also been found [13]. In our study, a more precise RACE technique was used to determine the length of RABV leRNA in the brain of mice infected with RABV DRV-AH08 and it was found that the length of leRNA varies from 40 to 79 nt, among which the most frequent size is 64 nt. To further validate the length of leRNA we extracted the total RNA from DRV-AH08-infected SK-N-SH cells at the early stage of infection, another method was used to construct leRNA library and to measure the length of different leRNA using high-throughput sequencing. Again, 64 nt long RNA was found to be one of the most prominent variants. These longer leRNA could be due to readthrough transcripts although the mechanism is not clear at the moment. The differences in leRNA lengths between the present and previous studies might be sue to differences in the methods used for detection. It is also possible, although unlikely, that different viruses used in these studies could explain the different results in leRNA length. In previous studies the predominant leRNA species of 56 or 58 nt was detected in cells infected with lab-adapted RABV. In the present study, the predominant 64 nt leRNA was detected in mice and cells infected with a wild-type RABV. Future studies are warranted to investigate these differences.

It has been reported that leRNA plays an important role in regulating RABV replication and transcription [22]. LeRNA is encapsidated by RABV N protein, preventing RABV from further initiating genomic RNA transcription [26]. Here we demonstrated, for the first time, that leRNA (direct transfection or as delivered by AAV vectors) can inhibit RABV replication both in cell culture and in infected animals. All the leRNA species has similar secondary structures despite of the differences in lengths and showed similar effects on RABV replication. Although leRNA can inhibit RABV replication via activating RIG-I-mediated immune responses, as double-stranded RNA and 5′-triphosphate RNA from viruses can be recognized in the cytoplasm by RIG-I, RLRs (RIG-I-like receptors), and Mda-5 [20, 41, 42], the observed inhibitory effects by leRNA in the present study seem to be more direct. Over-expression of leRNA not only reduced the total, but also the encapsidated genomic RNA. It is most likely due to leRNA-mediated interference of RABV genomic RNA, N and P ribonucleoprotein (RNP) complex, which are essential for RABV transcription and replication [18, 43, 44]. As it has been shown that N protein binds preferentially to leRNA compared to mRNA and non-viral RNA. It would be conceivable that over-expression of leRNA can override genomic RNA binding to N protein [26, 45]. The amount of leRNA was indeed elegantly upregulated, possibly to avoid virus over-replication which could induce premature death of the host cells during the early stage. During the late stage, leRNA was downregulated, which promotes genome replication. Similar phenomenon (autoregulation of virus replication) has also been found in other viruses, such as DENV–vsRNA-5 from DENV, miR-BART2 from Epstein-Barr virus (EBV) [10, 46]. These findings suggest RABV leRNA plays a pivotal role in regulating virus replication to avoid over-replication of the virus during the early stage of infection.

In the present study, a host protein, Hsc70, has been identified to interact with RABV leRNA. Using tRSA aptamer-mediated RNA-binding protein precipitation experiment and EMSA assay, it is demonstrated that leRNA directly binds to Hsc70. The RNA-Protein complex modelling suggests that the 51–59 nt of leRNAs are likely packaged by a positively charged amino acids rich N-terminal nucleotide-binding pocket (NBD) in Hsc70. Interestingly, the sequence from 51- 59 nt of leRNA was an AU rich domain, consistent with previous data showing that Hsc70 binds to AU-rich elements (AREs) [30]. As Hsc70, Hsp70 and Hsp60 all belong to Hsp70 family, Hsp60 and Hsp90 may also interact with RABV leRNA. Notably, our data demonstrate that Hsc70 is crucial for the inhibitory effect of leRNA on RABV replication, as knockdown of Hsc70 enhanced this effect of leRNA. In this line, the expression of Hsc70 is significantly down-regulated during the early stage and gradually upregulated during the late stage of RABV infection. In reminiscence of the amount of leRNA during infection, which was upregulated in early stage and gradually down-regulated during the late stage. Thus we hypothesize that Hsc70 plays a positive role in RABV replication by down-regulating leRNA. Accordingly, when Hsc70 is knocked down, the amount of leRNA is significantly upregulated while virus replication decreases. Although Hsc70 and RABV N interacts with leRNA [26], it is unlikely that N and Hsc70 compete for leRNA since Hsc70 knock-down increased N, P gene expression (Supplementary Figure 3) and suppressed viral replication. It has been shown that Hsc70/Hsp90 chaperone machinery is required to load small RNA duplexes into Argonaute proteins [34, 47]. Argonaute 2 (AGO2) is associated with both miRNA and RNAi pathway, and is able to cleave pre-miRNA to mature miRNA [25, 46]. Moreover, it has been demonstrated that AGO2 could be used to produce functional miRNA-like viral small RNA derived from RNA viruses [10]. Thus, it would be of great interest to further investigate whether Hsc70 and AGO2 pathway is involved in the cleavage or degradation of leRNA. Meanwhile Hsp70 (also known as HSPA1A) has been shown to interact with RABV N protein and regulate RABV infection [48]. It is conceivable that Hsc70, Hsp70, N protein and leRNA may form a complex and modulate RABV replication and transcription.

Since over-expression of leRNA specifically inhibits RABV replication *in vitro* and *in vivo*, the potential to use leRNA as a novel drug in rabies post-exposure prophylaxis was determined. Currently a combination of rabies vaccination and rabies immunoglobulin was given to people severely exposed to rabies according to WHO recommendations [49]. However, anti-rabies immunoglobulins are in short supply and too expensive [50]. RNA-based biopharmaceuticals are new class of therapies demonstrating significant growth and potential for the treatment and prevention of chronic and serious diseases [51–53]. Compared to immunoglobulins, leRNA is readily available and inexpensive and thus would be potentially helpful in assisting rabies post-exposure prophylaxis. In our study, AAV was applied to transcribe leRNA after exposure with DRV-AH08, which resulted in reduction of mortality in mice treated with leRNA than scrambled RNA. Although the success is somewhat limited considering the slow rate of gene transcription and expression *in vivo* by *AAV* vector, it could be further optimized by increasing the leRNA at the site of infection such as direct electroplating leRNA.

## MATERIALS AND METHODS

### Plasmids construction

The sequence of Hsc70 shRNA (GGTGTGCTT ATTCAGGTTTATGAAGCTTGATAAACCTGAATAAG CACACC), shGFP (TTGATGCCGTTCTTCTGCTTGT CGAAGCTTGGACAAGCAGAAGAACGGCATCAA), scrambled leader RNA (AGGACTCTATGTATTAGCA CAAGAACAACTAACTATCAAACGTAAAGACAAG AGGATCAAACAC) and leRNA (56 nt, 58 nt and 64 nt) were constructed into pAAV-U6 vector containing U6 promoter and mCherry reporter to obtain pAAV-U6-shHsc70, pAAV-U6-shGFP, pAAV-U6-scrambled RNA and pAAV-U6-leRNA plasmids. The RNA bait was cloned into pcDNA3-tRSA (general gift from Dr. Ian G. Macara, University of Virginia School of Medicine, USA) and then *in vitro* transcribed to obtain bait RNA which is used in RNA binding protein pull-down assay. The DRV-AH08 N protein with Fc tag and P protein were cloned into pcDNA-3.1, respectively. The genome fragment (1–1514 bp) was also cloned into pcDNA-3.1 flanked by hammerhead ribozyme (HamRz) and hepatitis delta virus ribozyme (HdvRz) sequence cDNA.

### Cell culture

Mouse neuroblastoma (NA) cells and HEK-293T cells were cultured in RPMI 1640 medium (Gibco) containing 10% fetal bovine serum (FBS, Gibco) at 37°C with 5% CO_2_. Human SK-N-SH cell line was grown in Minimum Essential Medium (MEM, Gibco) supplemented with 15% FBS.

### Virus production and titration

DRV-AH08 is a wild type rabies virus (RABV) isolated from a pathogenetic dog in Anhui province, China [54]. Virus stocks were prepared in 1-day-old suckling mice as previously described [55]. 96-well plates were inoculated with serial 10-fold dilutions of the virus. All titrations were inoculated with 20 000 NA cells/well and incubated at 37°C for 48 h, which were carried out in quadruplicate. The cells were fixed with 80% ice-cold acetone for 30 min, and then were stained using FITC-conjugated anti-RABV N monoclonal antibodies (1:120, Fujirebio) after washing with PBS. Antigen-positive foci were counted under a fluorescence microscope (Olympus, Japan). The virus titer was calculated as focus-forming units per milliliter (FFU/ml). Adeno-associated virus (rAAV) was produced in HEK-293T cells by co-transfecting pAAV with Helper plasmids as previously described [56]. 96-well plates were inoculated with serial 10-fold dilutions of virus. All titrations were inoculated with 50 000 HEK-293T cells/well and incubated at 37°C for 48 h, which were carried out in quadruplicate. The infectious titer of rAAV was determined using red fluorescent protein (mCherry), and each red cell under fluorescence microscopy represents one IU.

### Real Time PCR

Total RNA were extracted from cells using TROZIL (Invitrogen). The cDNA of leRNA and viral genomic RNA (vRNA) were reversely transcribed by AMV reverse transcriptase (Takara) and specific primers. For quantification, 1 μl of cDNA, 5 pM of primers and SYBR^®^ Select Master Mix kit (Life technologies) were used. For the quantification of leRNA, cDNAs were synthesized with tagged primer (CCAGATGCTTGGCGTCCTGCTTTA CAAATGACGCTGTC) with an 18-nucleotide (nt) tag that was unrelated to RABV. qRT-PCR was performed using primers leRNA-F (CCAGATGCTTGGCGTCCT) and leRNA-R (ACGCTTAACAACAAAATC). However, these primers could also amplify the antigenomic RNA, of which the level is negligible compared to that of genomic RNA [57]. For the quantification of vRNA, cDNAs were synthesized with tagged primer (GGAAGCATTTGTC CCCGATGGAAAAGGGACGTTTGAAAGGA) with a 19-nucleotide (nt) tag that was unrelated to RABV. qRT-PCR was performed using primers vRNA-F (GGAAG CATTTGTCCCCGAT) and vRNA-R (CAATTCAGCCG CCTCATA). For normalizing data of leRNA and genomic RNA level, U6 small nuclear RNA was used, GAPDH was used to normalize data of Hsc70 expression level.

### *In vitro* synthesis of scrambled RNA and leRNA

The sense and anti-sense scrambled RNA and leRNA were synthesized from Genescript (China) and cloned into pcDNA-3-T7 vector after annealing. The plasmids were linearized by enzyme digestion, and then subjected to *in vitro* transcription using AmpliScribe™ T7-Flash™ Transcription Kit (Epicentre Biotechnologies). The *in vitro* transcribed RNAs were checked by electrophoresis and spectrometry, and then stored at –80°C.

### RNA binding protein pull-down assay

The RNA binding protein pull-down assay was performed as described [35]. Briefly, 30 μg of *in vitro* synthetic RNAs were denatured at 65°C for 5min, and then applied to 50 μl streptavidin beads (Thermo) in 800 μllysis buffer (RIPA lysis buffer, Beyotime) with protease inhibitor (Roche) and RNasin (200 U/ml, Promega) after cooling to room temperature. The mixer were incubated on a rotation shaker at 4°C for 2–2.5 h. Total mouse brain lysate was collected by centrifuging at 4°C for 10 min at 16,000 g after grinded in lysis buffer (RIPA lysis buffer, Beyotime) with protease inhibitor (Roche) and RNasin (200 U/ml, Promega). The supernatants were incubated with Yeast RNA (Sigma) and Egg white avidin (EMD chemicals) to block endogenous biotinylated proteins and non-specific RNPs, and were cleared by centrifuge. Next, the cleared lysates were incubated with RNA-beads in a new tube on a rotation shaker at 4°C for 2.5–3 h. The supernatant was discarded after centrifugation for 2 min at 4°C. After washing with fresh lysis buffer for 5 times, the captured proteins were eluted and analyzed by SDS–PAGE with silver staining. The specific RNA binding protein bands were cut from the gel and subjected tocommercial mass spectrometry analysis (Shanghai applied protein technology, China).

### RNA immunoprecipitation

N protein with Fc tag, P protein and DRV-AH08 genomic fragment (1–1514 bp) (Described above) were co-expressed with the leRNA or control RNA in HEK-293T cells. The cells were harvested by trypsinization and resuspended in RIPA buffer (with 200 U/ml RNase inhibitor and protease inhibitor), kept on ice for 20 min (with frequent mixing). Centrifuged at 4°C, 10000 g for 10 min. Take 100 μlof the supernatant for the detection of input protein and input RNA. The proteins in supernatant were treated with protein A/G (a recombinant fusion protein that combines IgG binding domains of both Protein A and Protein G) beads (40 μl) (Santa cruz) and incubated for 2 h at 4°C with gentle rotation. The unbound material (proteins) was washed- off for 5 times by RIPA buffer. Isolate N protein coprecipitated RNAs by resuspending beads in TRIZOL RNA extraction reagent (1 ml). Reverse transcription of DNase treated RNA to cDNA and analysis by qRT-PCR.

### Non-coding RNA overexpression

For shRNA, scrambled leader RNA and leRNA, the sense and anti-sense ssDNA oligonucleotides were synthesized and inserted into pAAV-U6 after annealing. The AAV virus rAAV-U6-shRNA and rAAV-U6-leRNA were produced in HEK-293T cells as previously described [56] to express shRNA and leRNA *in vitro* and *in vivo*. Three kinds of control RNAs for leRNA with hairpin structure but no homologous with the genome of human, mouse and rabies virus, had been used. One of the control RNAs (CACATGAAGCAGCACGACTTC TTGAAGCTTGAAGAAGTCGTGCTGCTTCATGTG) has been cloned into pAAV-U6 to get pAAV-U6-cotrol RNA plasmid and rAAV-U6-cotrol RNA AAV virus, one of the control RNAs (UUCUCCGAACGUGUCACGUAC GUGACACGUUCGGAGAA) has been synthesized and used in transfection, and another control RNA was scrambled leader RNA (AGGACTCTATGTATTAG CACAAGAACAACTAACTATCAAACGTAAAGACAA GAGGATCAAACAC) has been cloned into pAAV-U6 to get pAAV-U6-scrambled RNA plasmid and used in transfection.

### Electrophoretic mobility shift assay (EMSA)

Hsc70 and the control protein 4BOW with 6x His tag were expressed in prokaryotic expression system and purified. LeRNA was incubated with Hsc70 and 4BOW respectively for 30 min at room temperature in binding buffer (50 mM Tris pH 7.5, 100 mM NaCl, 25 mM MgCl_2_, 20 mM DTT, 50% v/v glycerol, 0.5 mg/mL BSA). Electrophoresis was carried out on 8% w/v polyacrylamide non-denaturing gel in 0.5 × TG buffer (40 mMTris-base, 2.5 mM glycine). Gels were stained using SYBR^®^ Gold Nucleic Acid Gel Stain (1:10000, Life technologies). Scrambled RNA was also incubated with Hsc70 for 30 min at room temperature in binding buffer too.

### Immunohistochemistry

Cells infected with DRV-AH08 were fixed with 80% ice-cold acetone for 30 min at room temperature, Then cells were incubated with FITC conjugated anti-rabies monoclonal antibody (1:120, FUJIREBIO) for 1 h at 37°C after washing for three times with PBS. Adult mice were anesthetized and perfused transcardially with 4% PFA/PBS. The brains were dissected out and post-fixed in 4% PFA in PBS overnight at 4°C. Brain sections (30μm) were prepared with vibratome, and then incubated with mouse anti-P monoclonal antibody in PBS with 0.2% Triton X-100 and 1% BSA at 4°C overnight. Signals were developed by incubating sections with Alexa Fluor^®^ dye-conjugated IgG secondary antibodies (1: 400, Life technologies) for 4 h at 37°C in PBS. Images were obtained by epifluorescence microscope (Olympus, Japan).

### Animal experiments

The Control RNA-expressing rAAV and leRNA-expressing rAAV were stereotactically injected to the mouse hippocampus or intramuscularly injected to the mouse hind limb. Rabies virus (DRV-AH08) was injected intramuscularly. Mice were anaesthetized and perfused with PBS, and brains were fixed with 4% paraformaldehyde (PFA). All of the animal experiments were approved by the Research Ethics Committee, Huazhong Agricultural University, Hubei, China (HZAUMO2015-0016) and in accordance with the Guide for the Care and Use of Laboratory Animals from Research Ethics Committee, Huazhong Agricultural University.

### RNA structure analysis

RNA fold webserve was used to build the leRNA secondary structure. After the sequence of leRNA was imported to the Webserver, the suggested minimum free energy RNA secondary structure was returned (http://rna.tbi.univie.ac.at/cgi-bin/RNAfold.cgi).

### Statistical analysis

The significant differences of virus titer, protein expression, RNA levels, and mean fluorescence intensity were analyzed using Student *T-test*, one-way or two-way ANOVA by GraphPad Prism software (version 5.01). The statistical significance of survival rates was determined by the log-rank test and Kaplan-Meier survival analysis. Differences were considered statistically significant when *P <* 0.05.

## SUPPLEMENTARY MATERIALS FIGURES AND TABLES


