# Supplementary material for: Rabies viruses leader RNA interacts with host Hsc70 and inhibits virus replication
Source: Oncotarget. 2017 Mar 23;8(27):43822–37. doi: 10.18632/oncotarget.16517 (PMC5546443; doi:10.18632/oncotarget.16517)
Supplement: Supplementary file 1 [file oncotarget-08-43822-s001.pdf]

Rabies viruses leader RNA interacts with host Hsc70 and inhibits virus replication

Supplementary Materials

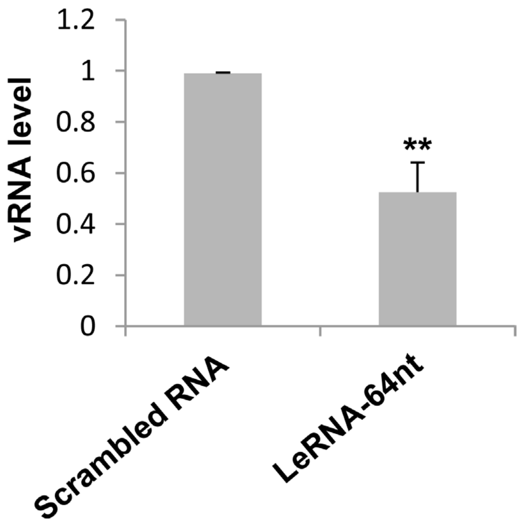

Supplementary Figure 1: SK-N-SH cells were infected with rAAV-U6-leRNA-64 nt AAV virus or rAAV-U6-scrambled RNA AAV virus, and after 24 h infected with DRV-AH08 (MOI is 0.01). The levels of DRV-AH08 viral genomic RNA (vRNA) were detected by Real Time PCR at 30 h post-infection with DRV-AH08.

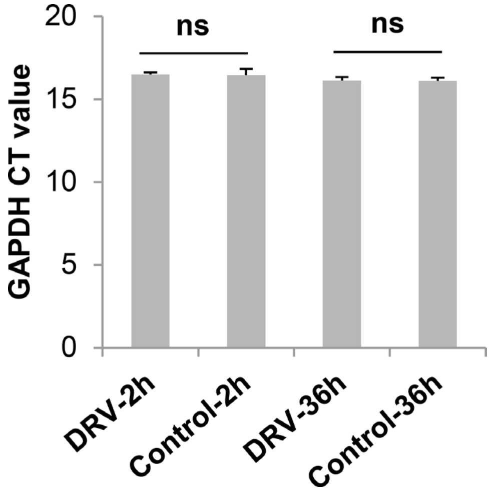

Supplementary Figure 2: The CT value of GAPDH at different time points post-infection. Total RNA of cells infected with DRV-AH08 or control cells were extracted at 2 h and 36 h post-infection, following by reverse transcription using same amount of total RNA. Then, the same amount of cDNA was used for Real Time PCR to detect the CT value.

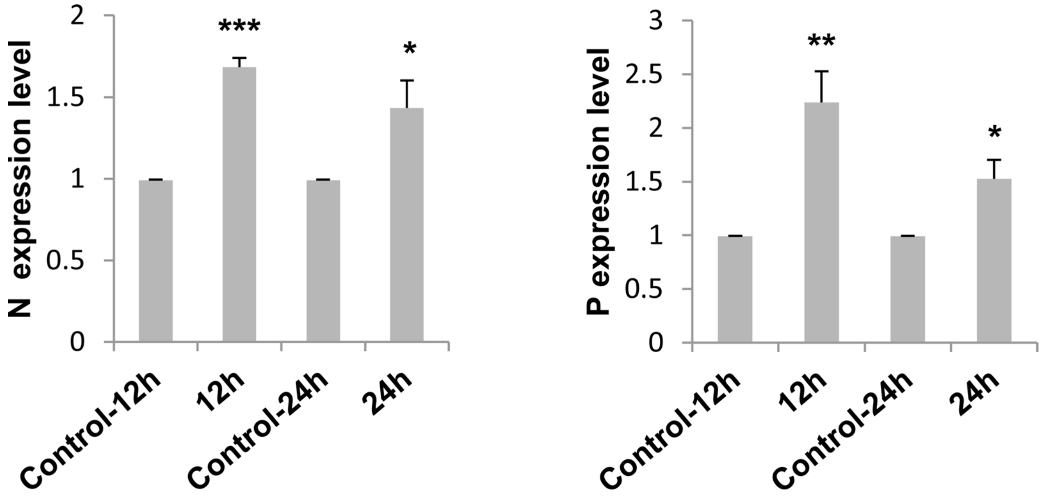

Supplementary Figure 3: SK-N-SH cells were transfected with shGFP (Control) or shHsc70. After 24 h, cells were infected with DRV-AH08 (MOI is 0.01), 12 h and 24 h post-infection with DRV-AH08 cells were analyzed by Real Time PCR.
